# Preferences of *Coptotermes formosanus* Shiraki and *Coptotermes gestroi* (Wasmann) (Blattodea: Rhinotermitidae) among Three Commercial Wood Species

**DOI:** 10.3390/insects2040499

**Published:** 2011-11-25

**Authors:** Nirmala K. Hapukotuwa, J. Kenneth Grace

**Affiliations:** College of Tropical Agriculture & Human Resources, University of Hawaii at Manoa, 3050 Maile Way, Gilmore Hall 310, Honolulu, HI 96822, USA; E-Mail: kennethg@hawaii.edu

**Keywords:** *Coptotermes formosanus*, *C. gestroi*, feeding preferences

## Abstract

The Formosan subterranean termite, *Coptotermes formosanus* Shiraki, and the Asian subterranean termite, *Coptotermes gestroi* (Wasmann), are both pests of wood in service in Hawaii and Florida. We conducted a laboratory study using method modified from those described in standard E1-09 of the American Wood Protection Association (AWPA 2009) to assess the termite resistance of three commercially available wood species used in regions of the USA where both termite species occur: Douglas fir, *Pseudotsuga menziessii*, southern yellow pine, *Pinus* spp. and redwood, *Sequoia sempervirens*. A multiple-choice (three-choice) assay was used for four weeks (28 days) in order to simulate field conditions of food choice and assess termite feeding preferences under 28 °C and 72–80% RH. 400 termites (360 workers: 40 soldiers) were released into each test jar. Five replicates and two controls of each wood species were used with each termite species. Termite mortality was recorded at the end of the test; and wood wafers were oven-dried and weighed before and after termite exposure to determine the mass loss due to termite feeding, and rated visually on a 0 (failure) to 10 (sound) scale. There were significant differences in mean mass loss values among the three wood species and between two termite species. The mean mass loss value for redwood was significantly lower than Douglas fir and southern yellow pine with both termite species. However, *C. formosanus* showed increased feeding on Douglas fir and southern yellow pine compared to *C. gestroi*.

## 1. Introduction

Termite feeding activities play an important role in nutrient and energy cycles in ecosystems [1]. Numerous experiments have been conducted to study their foraging behavior, feeding preferences, feeding rates (wood consumption rates), *etc*. One unique aspect of subterranean termite foraging is that they must locate food in soil by constructing underground tunnels [2]. Like other social insects, subterranean termites have very efficient searching systems [3] consisting of branching gallery systems, with the architecture and speed of construction of the system varying among species. These gallery systems have been excavated and have been mapped by different researchers using laboratory foraging arenas. Some of the findings of these studies have been: the tunnel distribution of the eastern subterranean termite *Reticulitermes flavipes* (Kollar) was optimized for food searching efficiency [3]; food size affected tunnel volume and length of tunnels of the Formosan subterranean termite, *C. formosanus* Shiraki [4]; the presence of food did not impact tunnel distribution of *C. formosanus* [5,6]; tunneling activity increased towards a positive moisture gradient [7]; sand moisture has significant effect on termite distribution and food consumption rates whereas soldier proportions had no effect [8]; the amount of wood consumed generally increased with the increase in drying temperature and heat contributed to the loss of natural resistance components of wood [9]; and that workers of large body weight *C. formosanus* consumed a minimum rate of wood while workers of smaller weight consumed a higher rate of wood [10]. In the latter study, termite feeding rates were also reported to increase with an increase in soldier proportion to 30% [10].

Furthermore, wood preferences of termites have been extensively studied. Some examples are: natural resistance of Alaska cedar, redwood and teak to Formosan subterranean termites [11]; wood consumption rates and survival of the Formosan subterranean termite against six commercially used woods (Douglas fir, Hemlock, spruce, Ponderosa pine, Cedar and Redwood) in Hawaii [12]; wood preference of selected Malaysian subterranean termites [13]; wood consumption rates of forest species by subterranean termites under field conditions [14]; comparative study of two Pakistan subterranean termites for natural resistance and feeding preference in laboratory and field trials [9]; effect of cellulose concentration on the feeding preferences of the termite *Reticulitermes flavipes* [15]; resistance of eastern red cedar panels to damage by subterranean termites [16]; survival and feeding response of *Anacanthotermes ochraceus* against local and imported wood [17]; natural resistance of some imported wood species to subterranean termites in Saudi Arabia [18]; evaluating the natural durability of native and tropical wood species against *Reticulitermes flavipes* [19]; Formosan subterranean termite feeding preference as basis for bait matrix development [20]; resistance of the Indonesian woods, Bangkira (*Shorea laevis*) and Merbu (*Intsia palembanica*) to Formosan subterranean termite attack [21]; and wood preferences of the Turkestan termite *Anacanthotermes turkestanicus* [22].

However, there have been few comparative examinations of the feeding preferences of *Coptotermes formosanus* and *Coptotermes gestroi*. Grace *et al.* [23] reported that the tunnel systems of *C. gestroi* are thinner and more highly branched than those of *C. formosanus*. The feeding rate of *C. gestroi* was also found to be lower than the feeding rate of *C. formosanus* [24].

The purposes of our study were to compare feeding rates and wood feeding preferences of these two termite species using a multiple choice test modified from AWPA (2009). Comparing wood preferences is useful for selecting building materials for use under conditions of high termite hazard, and to identify wood species requiring preservative treatment before use.

## 2. Materials and Methods

A three-choice, or multiple choice test modified from that described in Standard E1-09 of the American Wood Protection Association (AWPA 2009) was used to assess termite preferences for three different wood species used in Hawaii or in Florida, USA; regions where both termite species are found. Douglas fir, *Pseudotsuga menziessii,* is the principal wood used in building construction in Hawaii [12] and is highly susceptibility to termites. Southern yellow pine, *Pinus* spp., is commonly used for research purposes in laboratory termite evaluations, is a primary construction timber in Florida and along the Gulf Coast of the continental USA, and has low resistance to termites and decay. Redwood, *Sequoia sempervirens*, is a naturally durable, insect resistance timber. It is mainly used for decking, fencing, and other exterior applications, and is relatively expensive compared to other lumber.

### 2.1. Wood Blocks

Test samples were cut from each wood species with a band saw. Each test wafer was approximately 2.5 cm by 2.5 cm by 0.4 cm. All wood wafers were autoclaved (Getinge Auto Clave, Gettings USA, Inc, New York, NY, USA) at 256 °C and 20 PSI for 60 minutes to remove any molds. For each wood species there were five replicates and three environmental controls (exposed to the same test conditions, but without termites). All samples were dried in an oven (calibrated with a Salvis thermometer) at 90 °C for 24 hours. The samples were allowed to cool to room temperature in a desiccator for one hour, and weighed (Mettler AE 163).

### 2.2. Test Design

**Figure 1:**
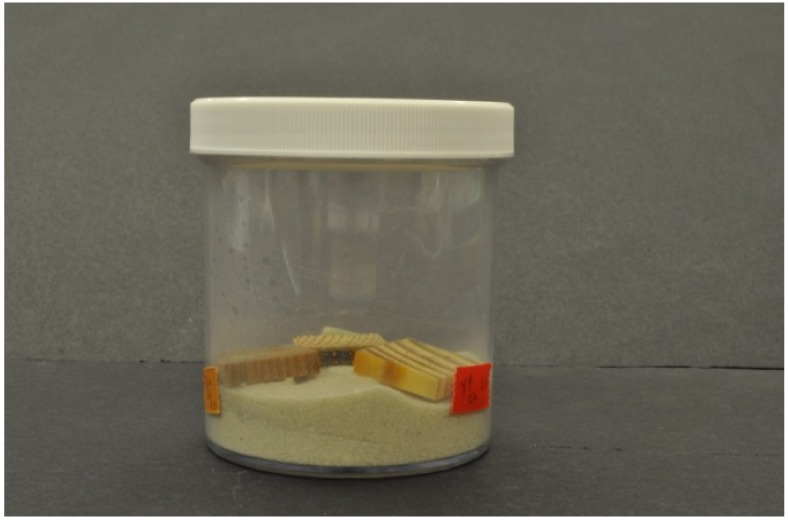
Sample test jar with three different wood species.

The test containers consisted of 85 mm diameter by 97 mm tall polystyrene jars with a plastic screw top lid. Two sets of jars were used: test jars (with live termites) and control jars (without termites). Each jar contained 150 g of silica sand (fine granules of 40–100 mesh, Fisher Scientific, Fair Lawn, New Jersey 07410), 30 mL of distilled water, and one wood wafer from each of the three different wood species (three wafers in the same jar–Figure 1).

### 2.3. Bioassays and Termites

Termites were collected from two different field sites: *C. gestroi* from Kalaeloa (formerly Barber's Point Naval Air Station) on the south-west side of the Island of Oahu, Hawaii, and *C. formosanus* from colonies located on the campus of the University of Hawaii at Manoa. Termites were collected using techniques modified from those of Tamashiro *et al.* [25] and Su and Scheffrahn [26]. Four hundred live termites (360:40, workers:soldiers) were released into each test container. The jar tops were replaced loosely. The jars were placed in an unlighted incubator at 28 °C and 72–80% RH for four weeks and were inspected weekly.

At the end of the experiment, all of the live termites were counted. All of the jars were disassembled and the blocks were removed. All blocks were allowed to air dry at room temperature for 24 hours and then oven dried at 90 °C for 24 hours. Next, they were allowed to cool to room temperature in a desiccator for one hour. Finally all the blocks were reweighed to determine the amount consumed. Each block was examined and visually rated using a standard method (AWPA 2009), which is described in Table 1. To compare termite feeding rates on the three different wood species, two-way ANOVA and Ryan-Einot-Gabriel-Welsch Multiple Range Test [REGWQ] were executed using SAS 9.2.

## 3. Results and Discussion

**Figure 2:**
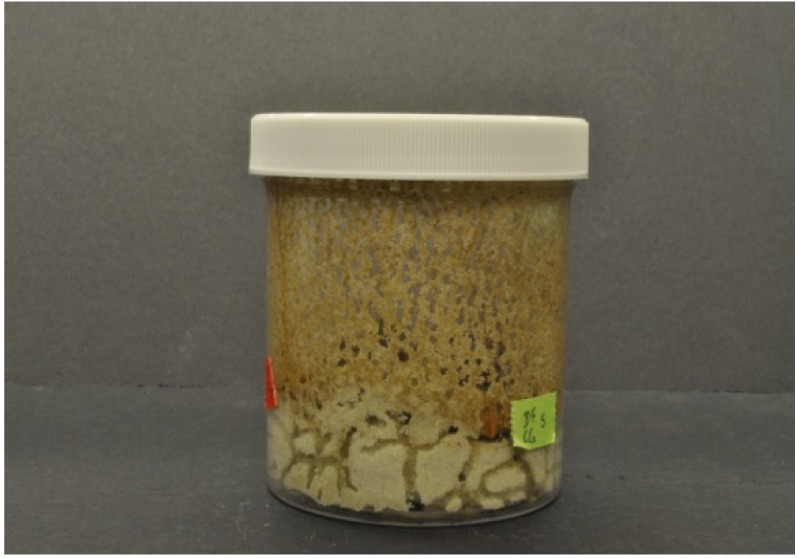
Tunnel network of *C. gestroi*.

**Figure 3:**
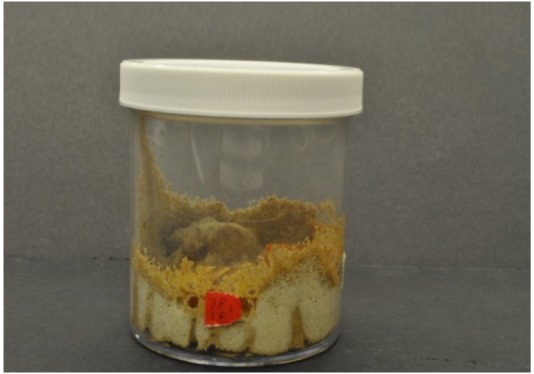
Tunnel network of *C. formosanus*.

**Figure 4:**
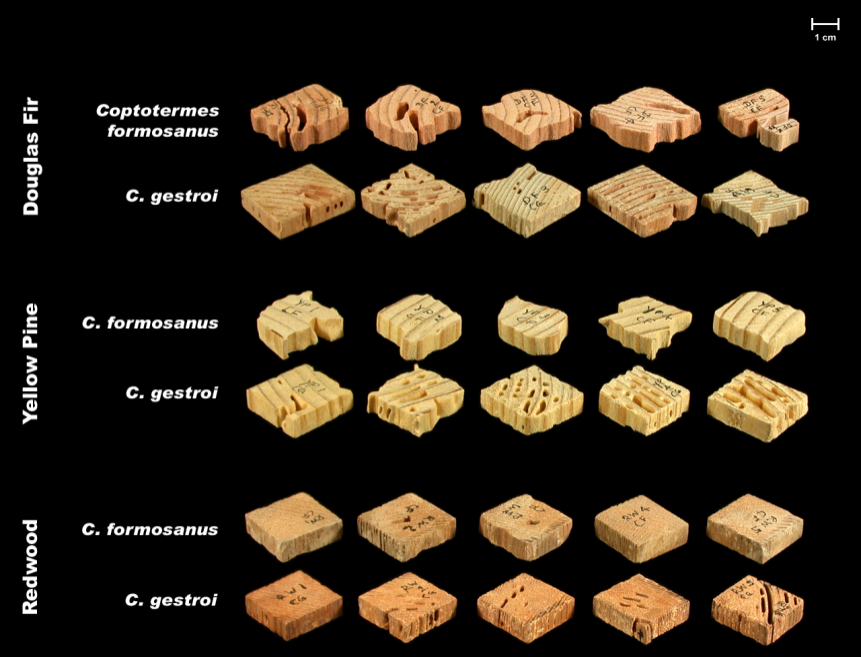
Extent of feeding by the two termite species on three wood species.

**Figure 5:**
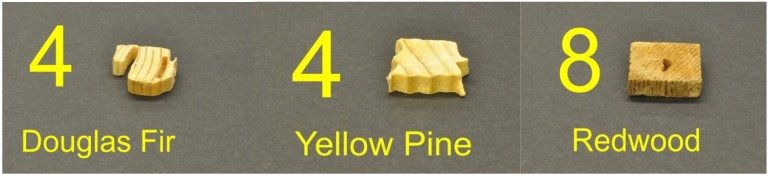
Sample visual ratings for *C. formosanus* EI Rating (modified from E1-09): 4 = very severe, 50–75% affected; 8 = moderate, 3–10% affected.

**Figure 6:**
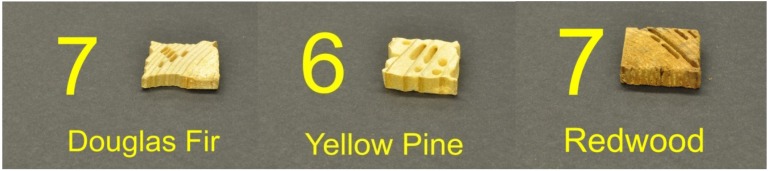
Sample visual ratings for *C. gestroi* EI Rating: 6 = severe, 30–50% affected; 7 = moderate/severe, 10–30% affected.

Visual observations supported the differences between the two species in their tunnel networks noted by Grace *et al.* [23]. *Coptotermes gestroi* made large numbers of narrow, highly branched tunnels; whereas *C. formosanus* made fewer, wider, and less branched tunnels (Figures 2 and 3). During the first weekly inspection period we observed that both species of termites contacted all types of wood, and some moved to the bottom of the jars and began to make tunnels. Also, both species began to feed upon Douglas fir and southern yellow pine. We also noted that *C. gestroi* made tunnels all the way to the top of some jars, while *C. formosanus* made a small number of tunnels, extending only halfway up the jars. During the second inspection period, most jars with *C. gestroi* were filled with sand tunnels, and some of the wood blocks of all three species were covered completely with sand. This made it difficult to accurately record the sequence of feeding patterns and consumption rates. With *C. formosanus*, we observed that some jars contained tunnels reaching all the way to the top, but jars were not as extensively tunneled as with *C. gestroi. Coptotermes formosanus* excavated fewer, wider tunnels and it was difficult to estimate feeding rates by looking through those jars. Only one block of redwood was covered completely with sand. During the third week, almost all of the jars with *C. gestroi* were covered completely with large numbers of tiny, highly branched tunnels; whereas *C. formosanus* made few long tunnels with less branching in 60% of all jars. Also, *C. formosanus* covered most of the redwood blocks with sand, while *C. gestroi* did not cover the redwood blocks. In all wood blocks attacked by *C. formosanus* we noted that they fed mostly on the outer parts (very slightly in redwood). *Coptotermes gestroi*, however, mostly fed on middle parts of the wood blocks, and they made a larger number of small holes and small narrow tunnels in all blocks (Figures 4, 5 and 6).

We also observed some differences in termite feeding rates. *Coptotermes formosanus* showed moderate feeding on Douglas fir and southern yellow pine, whereas *C. gestroi* showed light feeding rates for these two kinds of woods. With redwood, both termite species demonstrated either no apparent feeding or very light feeding. Overall, both species exhibited relatively high activity rates and feeding rates during the first, second and third weeks, but not during the fourth week. During the fourth (last) week, they exhibited slow motion, and slow feeding rates. All wafers without termites (environmental control jars) had very little fungal growth.

We observed some major differences in mean visual ratings and mean mass loss values among the three wood species (Table 1). In terms of visual ratings, both termite species showed severe attack on Douglas fir and southern yellow pine; whereas on redwood, *C. gestroi* showed moderate/severe attack but *C. formosanus* exhibited moderate attack. Mean wood mass loss values for *C. formosanus* were higher for both Douglas fir and southern yellow pine than those for *C. gestroi*. These finding are similar to those of Uchima and Grace [24]. However, for redwood, *C. gestroi* showed a relatively higher feeding rate than *C. formosanus*. But compared to feeding rates on Douglas fir and southern yellow pine for both species, feeding rates on redwood were very low. However, Su and Tamashiro [12] noted that in the field, *C. formosanus* had a high feeding rate (25.2 mg/g/day) and severely damaged redwood.

Wood resistance levels can be categorized into four major classes based on both visual ratings of termite damage and wood mass losses (modified from Grace *et al.* [27]): resistant, moderately resistant, slightly resistant and susceptible. Resistant woods were visually rated as 9 or better, with mean mass losses not exceeding 5 percent; those in the moderately resistant category were rated above 7, with mean mass losses not exceeding 10 percent; slightly resistant woods were rated above 6, with mass losses not exceeding 20 percent; while those considered susceptible received a visual rating of 6 or less, and sustained mean mass losses greater than 20 percent. According to this scale, our test results with these three wood species can be categorized as follows: Both Douglas fir and southern yellow pine are susceptible to *C. formosanus*. However, redwood is resistant to attack by *C. formosanus*. Both Douglas fir and southern yellow pine are slightly resistant to *C. gestroi* whereas redwood is moderately resistant. It is important to note, however, that this scale was developed with *C. formosanus*, and that *C. gestroi* has a slower feeding rate [24].

Mean percent termite mortality for *C. formosanus* was 16.50 (±5.84) and for *C. gestroi* was 19.50 (±8.52). These mortality rates were not significantly different (*p* = 1.000). Mortality rates were likely uniformly low due to the presence of an adequate quantity of relatively susceptible wood in each test container.

**Figure 7:**
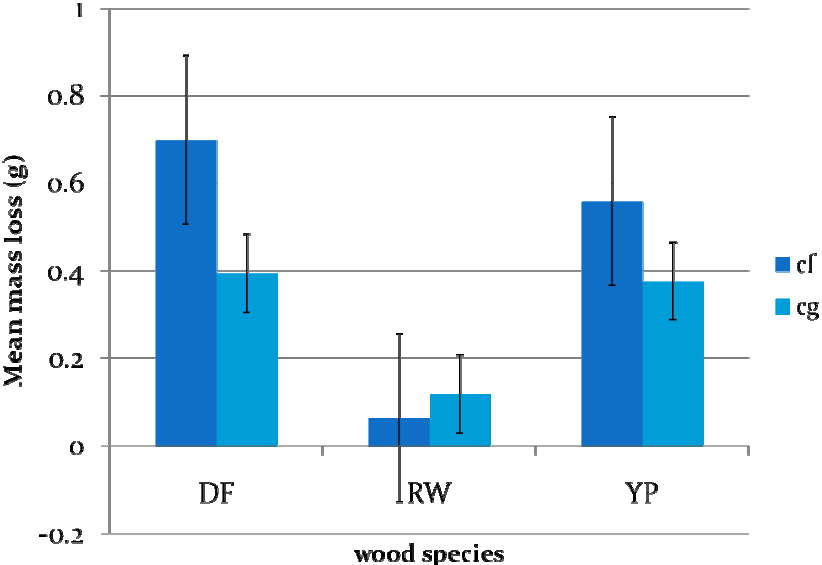
Mean mass loss of three wood species for *C. formosanus* and *C. gestroi*. (Two way ANOVA and Ryan-Einot-Gabriel-Welsch Multiple Range Test [REGWQ], P < 0.05) (DF = Douglas fir, YP = Southern Yellow pine, RW=Redwood *Cf = Coptotermes formosanus*, Cg *= Coptotermes gestroi*).

There were some significant differences in mean mass loss values among the three wood species (*F* = 38.81, *DF* = 2, *p* = 0.0001) and also between the two termite species (*F* = 10.22, *DF* = 1, *p* = 0.0039) (Figure 7, Table 2). The mean mass loss value for redwood was significantly different from those for Douglas fir and southern yellow pine. The overall mean wood mass loss value for *C. formosanus* was significantly different from that for *C. gestroi*, and *C. formosanus* showed higher feeding rates on Douglas fir and southern yellow pine than *C. gestroi*. These results are consistent with those of Uchima and Grace [24] with Douglas fir, and may reflect either size differences or differences in activity levels between the two *Coptotermes* species.

## 4. Conclusions

We found that feeding rates and wood preferences were slightly different between the two termites. Mean mass losses differed among the three wood species, and also between the two termite species. This is the first study to examine feeding preferences of these two *Coptotermes* species using commercial woods commonly used in either Hawaii or Florida, where both termite species occur. Results of studies of this type are important to determine the levels of preservative treatments required to protect timbers in regions with high termite pressure, and to identify naturally durable woods that may not require preservative treatment. Natural durability is an area of increasing interest due to the interest of policy makers in reducing migration of industrial chemicals into the environment [19].

## Figures and Tables

**Table 1:** Summary of results for *C. formosanus* and *C.gestroi* from multiple-choice test. Rating: 10 (sound), 9.5 (trace, surface nibbles permitted), 9 (slight attack up to 3% of cross sectional area affected), 8 (moderate attack, 3–10% of cross sectional area affected), 7 (moderate/severe attack, penetration, 10–30% of cross sectional area affected), 6 (severe attack, 30–50% of cross sectional area affected), 4 (very severe attack, 50–70% of cross sectional area affected) or 0 (failure).

**Wood Species**	**Termite Species**	**Mean Visual Rating**	**Mean Mass Loss (g)**	**Mean Percent Mass Loss**
Douglas fir *Pseudotsuga menziessii* (DF)	*Coptotermes formosanus* (Cf)	4.80 (±1.095)	0.6983 (±0.1668)	33.67 (±7.85)
Southern Yellow pine *Pinus* spp (YP)	C*optotermes formosanus*(Cf)	5.40 (±1.342)	0.5585 (±0.2130)	27.98 (±10.63)
Redwood *Sequoia sempervirens* (RW)	*Coptotermes formosanu*s(Cf)	8.6 (±0.548)	0.0639 (±0.0354)	4.75 (±2.73)
Douglas fir *Pseudotsuga menziessii* (DF)	*Coptotermes gestroi* (Cg)	6.1 (±1.342)	0.3947 (±0.0779)	13.39 (±9.52)
Southern Yellow pine *Pinus* spp (YP)	*Coptotermes gestroi (Cg)*	6.2 (±0.894)	0.3754 (±0.0909)	13.85 (±9.35)
Redwood *Sequoia sempervirens* (RW)	*Coptotermes gestroi* (Cg)	7.6 (±0.894)	0.1180(±0.0522)	6.28 (±4.78)

**Table 2:** Summary of results (Two-way ANOVA, Ryan-Einot-Gabriel-Welsch Multiple Range Test [REGWQ]) (SAS 9.2).

		**Mean Mass Loss (g)**
**Wood Species (*p* < 0.0001)**	Douglas fir *Pseudotsuga menziessii* (DF)	0.5465 a
	Yellow pine *Pinus spp* (YP)	0.4670 a
	Redwood *Sequoia sempervirens* (RW)	0.0910 b
**Termite Species (*p* = 0.0039)**	*Coptotermes formosanus*	0.4402 a
	*Coptotermes gestroi*	0.2960 b
